# Twenty-Five Years Experience on RET Genetic Screening on Hereditary MTC: An Update on The Prevalence of Germline RET Mutations

**DOI:** 10.3390/genes10090698

**Published:** 2019-09-10

**Authors:** Rossella Elisei, Alessia Tacito, Teresa Ramone, Raffaele Ciampi, Valeria Bottici, Virginia Cappagli, David Viola, Antonio Matrone, Loredana Lorusso, Laura Valerio, Carlotta Giani, Cristina Campopiano, Alessandro Prete, Laura Agate, Eleonora Molinaro, Cristina Romei

**Affiliations:** Department of Clinical and Experimental Medicine, Unit of Endocrinology University of Pisa, 56124 Pisa, Italy

**Keywords:** medullary thyroid carcinoma, genetic screening, *RET*, VUS

## Abstract

Background: Pathogenic germline mutations affecting the *RET* proto-oncogene underlie the development of hereditary medullary thyroid carcinoma (MTC). The aims of this study were to evaluate the prevalence of germline *RET* mutations in a large series of MTC, collected over the last 25 years, and to reappraise their clinical significance. Methods: We performed *RET* genetic screening in 2031 Italian subjects: patients who presented with sporadic (n = 1264) or hereditary (n = 117) MTC, plus 650 relatives. Results: A *RET* germline mutation was found in 115/117 (98.3%) hereditary and in 78/1264 (6.2%) apparently sporadic cases: in total, 42 distinct germline variants were found. The V804M mutation was the most prevalent in our cohort, especially in cases that presented as sporadic, while mutations affecting cysteine residues were the most frequent in the group of clinically hereditary cases. All M918T mutations were “de novo” and exclusively associated with MEN2B. Several variants of unknown significance (VUS) were also found. Conclusions: a) *RET* genetic screening is informative in both hereditary and sporadic MTC; b) the prevalence of different mutations varies with V804M being the most frequent; c) the association genotype–phenotype is confirmed; d) by *RET* screening, some VUS can be found but their pathogenic role must be demonstrated before screening the family.

## 1. Introduction

Medullary Thyroid Carcinoma (MTC) can be either sporadic or hereditary. The hereditary form gives origin to the Multiple Endocrine Neoplasia (MEN) disorders in which thyroid carcinoma can be associated with additional endocrine neoplasia such as pheochromocytoma and/or hyperparathyroidism (MEN2A) and with non endocrine diseases such as mucosal neuromas, megacolon, marfanoid habitus and skeletal abnormalities (MEN2B). A third form of hereditary MTC is represented by familial MTC (FMTC), which is characterized by MTC alone, but in several relatives of the same family [1,2]. The RET proto-oncogene encodes for a tyrosine kinase transmembrane receptor that regulates cell growth, proliferation, survival and differentiation [3]. The oncogenic transforming role of germline *RET* mutations in hereditary medullary thyroid carcinoma (MTC) has been reported 25 years ago [4]. Following the first description of point mutations in MTC the prevalence of these alterations and the correlation with different phenotypes have been extensively described and discussed in outstanding reviews [5,6,7]. In addition to the classical cysteine mutations, which were originally shown to be associated with the multiple endocrine neoplasia type 2A (MEN2A) [4] and the M918T mutation that is causative of MEN2B [8], over the years additional *RET* mutations have been discovered mainly in FMTC. These mutations occur mostly in cysteines, but can also affect non-cysteine codons, mainly at codons E768 [9], L790 and S791 in exon 13 [10], codon V804 in exon 14 [11] and codons A883 [12] and S891 in exon 15 [13]. Very rare *RET* mutations, of uncertain impact in disease pathogenesis (“variants of unknown significance”, VUS), have also been reported often in a single subject or family [14,15,16].

*RET* germline mutations have been found in almost all MEN2 families, with only about 2% of families affected by hereditary MTC having no detectable germline mutations [7,17]. *RET* germline mutations, mainly affecting non-cysteine codons, have been reported in about 5–10% of apparently sporadic MTC cases [18,19,20]. At the tumor level, somatic *RET* mutations, commonly point mutations but also deletions/insertions, have been described in about 50% of the sporadic MTC, according to data published in the COSMIC database [21]. In sporadic MTC, the presence of a somatic *RET* mutation, particularly M918T, has been found to correlate with more aggressive tumor features, a more advanced stage at diagnosis and a worse prognosis [22,23].

Although codon 634 mutations are the most prevalent mutations in both European and non-European families [24,25], mutations at codon 533 are dominant in Greece [26] and mutations at codon 618 are particularly frequent in Cyprus [27]. A recent large Italian study showed a high prevalence of codon 804 mutations [28] whereas a high prevalence of codon 790 mutations has been noted in Germany [10].

The introduction of *RET* genetic screening in clinical practice for hereditary MTC favorably modified the clinical management of MTC patients. According to the recent American [29] and European guidelines [30], all patients with MTC should undergo *RET* genetic screening to identify both hereditary cases erroneously diagnosed as sporadic and *RET* mutation carriers in MEN2 families. Relatives who are *RET* negative will not develop MTC, whereas *RET*-positive subjects are at risk to develop the cancer and must be followed and treated [29,31].

The aim of this study was to provide an update on the prevalence of germline *RET* mutations in our large series of sporadic and hereditary MTC after 25 years of genetic screening. A reappraisal of the clinical meaning of the *RET* mutations is also included.

## 2. Patients and Methods

### 2.1. Subjects

**Figure 1:**
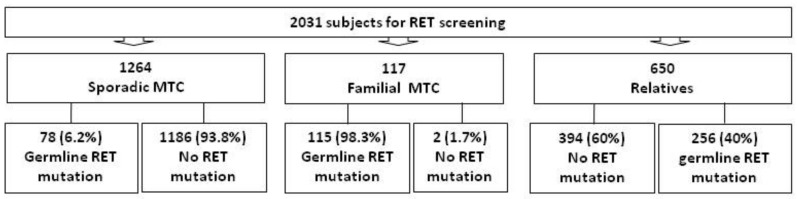
Subjects who underwent *RET* genetic screening over the last 25 years at our institution: the prevalence of *RET* mutations according to the first clinical presentations is reported.

During the last 25 years (1993-2018), we have performed *RET* genetic screening in a total of 2031 Italian subjects: 117 were clinically affected by a hereditary disease at diagnosis, 1264 were affected by a sporadic MTC form (i.e., no familial MTC history and no other endocrine diseases), 650 subjects were relatives of *RET* positive MTC patients (Figure 1). Informed consent for *RET* genetic screening and other clinical procedures was obtained from all investigated subjects. The present study has been approved by the local ethical committee.

### 2.2. RET Genetic Analysis

Blood has been collected over this 25-years period and stored at −20 °C. Over the years, the procedure of DNA extraction and sequencing analysis has been modified; until 2006, genomic DNA was purified from peripheral blood lymphocytes using the QIAMP blood kit (QIAGEN, Hilden, Germany). From 2006 until today an automated method (Maxwell16 Instrument, Promega, Madison, WI, USA) for genomic DNA extraction has been introduced. During this time interval also the *RET* sequencing has been modified: in the first years only exons 10, 11 and 16 were analyzed. Following the identification of additional *RET* mutation spread in a larger portion of the gene the analysis of exons 5, 8, 13, 14 and 15 has been introduced. With the exception of relatives of *RET* positive index cases, who have been analyzed only for the presence of the mutation identified in their family, all patients included in the study have been screened for the presence of *RET* mutations in eight exons (5, 8, 10, 11, 13, 14, 15 and 16). In familial cases with no evidence of *RET* mutations in these commonly analyzed exons, the entire coding sequence of the *RET* gene was analyzed. The biological rationale to focus on this region of the *RET* gene is that these mutations cause ligand-independent constitutive activation of the tyrosine kinase receptor. Cysteine mutations induce the formation of disulphide-bonded homodimers while mutations in the tyrosine kinase domain of the receptor, in particular the M918T mutation, cause a constitutive high level of autophosphorylation. Both mechanisms lead to activation of downstream signaling pathways, independent of ligand binding to the receptor.

Currently, genomic DNA is amplified using KAPA2G Fast HotStart PCR Kit (Sigma-Aldrich, Saint Louis, MO, USA) in a final volume of 25 μL (My Cycle instrument, Biorad, Hercules, CA, USA). Amplification cycle is performed with an initial step of 95 °C for 2 min, followed by 35 cycles of 95 °C for 15 s, 60 °C for 15 s and 72 °C for 15 s. A final extension at 72 °C for 7 min was performed at the end of the amplification protocol. Sequence analysis was performed and has been reported on previously [32]. Primers’sequences can be provided upon request.

## 3. Results

Among the 2031 subjects submitted to *RET* genetic screening in the period 1993-2018 (25 yrs), 117 were clinically affected by a hereditary disease at diagnosis: 115/117 (98.3%) were found to be carriers of a *RET* germline mutation, while two families (1.7%) had no detectable *RET* mutations. A germline *RET* mutation was also found in 78/1264 (6.2%) MTC patients who were diagnosed as sporadic according to their negative familial history and absence of other endocrine neoplasms. Considering the cases found to be familial at diagnosis (clinically familial cases) and familial cases erroneously diagnosed as sporadic (apparently sporadic cases) a total of 195 families with hereditary MTC were followed at our hospital. The remaining 1186 sporadic MTC patients were found to be negative for germline *RET* mutations and their sporadic nature has been confirmed.

Following the identification of a *RET* mutation in the index cases, 650 relatives have been analyzed and 256 (40%) subjects were found to carry the same *RET* mutation as their affected family members. The remaining 394 (60%) individuals were *RET* negative (Figure 1). In the whole series, 449 subjects have been found to harbour a *RET* germline mutations. *RET* mutations that are inherited as autosomal dominant Mendelian traits, were present in about 50% of family members. An exception to this prevision is represented by the MEN2B-associated M918T mutation, which was present in about 30% of our patients and was found to be a de novo germline mutation in all cases. In addition to the MEN2B patients, seven MEN2A-associated mutations were proven to be “de novo”. Five of these cases had a C634X, 1 had a C618S and the last a C620S germline mutation.

**Figure 2:**
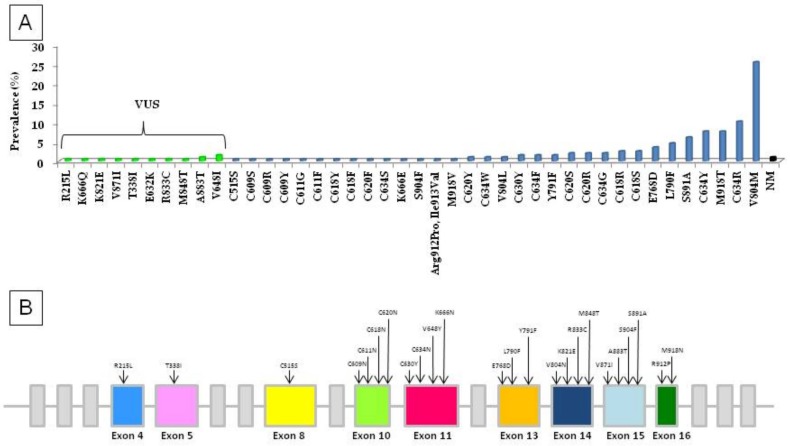
Prevalence (**A**) and distribution along the gene (**B**) of *RET* germline mutations in our series. In this series 11 mutations were variants of unknown significance (VUS).

All together 42 different germline missense mutations have been identified (Figure 2). The V804M *RET* mutation was the most prevalent missense variant in our series, both when considering all cases together (50/195, 25.6%) and when considering clinically familial (23/117, 19.6%) or apparently sporadic (27/78, 34.6%) cases. When taken together, mutations affecting the cysteine at codon 634 (C634R/Y/G/F/W/S) were found in 45/195 cases (23%). Cys634 mutations, were the most prevalent germline mutations in the group of clinically familial cases (37/117, 31.6%) and were significantly (*p* = 0.0005) more frequent in this group than in the group of apparently sporadic cases (8/78, 10.2%). When considering all cysteine mutations together a significantly higher prevalence was observed in clinically familial cases than in apparently sporadic cases (54/117 (46.1%) vs 23/78 (29.5%), respectively) (*p* = 0.02). Conversely, germline mutations affecting non-cysteine residues were more frequent in apparently sporadic than in clinically familial cases (55/78 (69.2%) vs 61/117, (52%), respectively) (*p* = 0.01). The M918T mutation, found in 15/117 (12.8%) cases, was always a de novo mutation and the patients were all affected by MEN2B syndrome.

**Figure 3:**
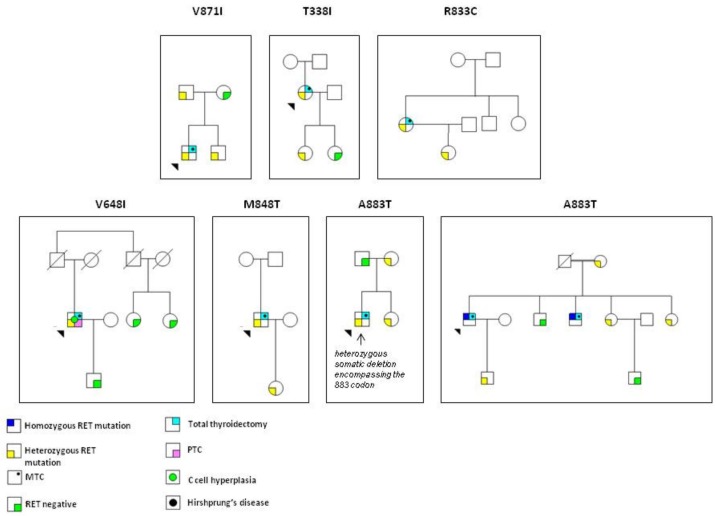
Pedigrees of MEN2 families with germline *RET* mutation classified as VUS. With the exception of a A883T *RET* mutated family in which medullary thyroid carcinoma (MTC) was present in two homozygous patients, MTC was present only in one member of the family. PTC: Papillary Thyroid Cancer.

Among all mutations, we found some rare variants (R215L, K666Q, K821E, V871I, T338I, E632K, V648I, R833C, M848T, A883T) previously reported but of unknown biological significance (VUS) (Table 1). The VUS were found in 3/117 (2.5%) cases of familial MTC and in 11/78 (14.1%) apparently sporadic cases. In 4 families these variants were found only in the index cases and in eight cases more than one family member was carrying the *RET* mutation but only the index case presented with MTC so far (Figure 3). Moreover, very rare germline synonymous mutations, which did not modify the encoded aminoacid (GAC->GAT, D631D; AAC->AAT, N763N; TTC->TTT, F893F) were found in three clinically sporadic cases and, so far, their role in MTC pathogenesis, if any, is undefined.

As shown in Table 2, among the 45 families with a C634 mutation in exon 11, 32 (71.1%) had PHEO (pheochromocytoma) and in six cases (13.3%) also hyperPTH (hyperparathyroidism). Among the families with a cysteine mutation in exon 10, PHEO or hyperPTH were present in 5/28 (17.8%) families. In the M918T cases affected with MEN2B, PHEO was present in 4/15 and Marphan syndrome and/or mucosal neurinomas were present in all cases while none of them had hyperPTH. Among families with non-cysteine mutation only two, both carrying a V804M mutation showed the association of MTC and PHEO in several family members. In a third V804M family, the association of MTC with hyperPTH was observed in one single individual. Three MEN2A families showed as association with the Hirshprungs’ disease and carried a *RET* germline mutation in exon 10 (C609R, C618R and C620Y).

## 4. Discussion

Soon after the discovery of the driving role of *RET* mutations in the pathogenesis of MTC [4,8] *RET* genetic screening in patients with hereditary and sporadic MTC has been introduced in the clinical practice to identify *RET* positive subjects and their relatives who are at high risk of developing MTC during their life.

Over the last 25 years, we have screened the *RET* gene in a series of 2031 Italian subjects including 1381 MTC patients and 650 relatives. As a standard routine procedure, only *RET* exons reported to be causative of the inherited MTC have been investigated (exons 5, 8, 10, 11, 13, 14, 15 and 16). Furthermore, in patients with familial MTC who were negative on the first screen, the whole *RET* coding sequence has been analyzed. In agreement with previously reported data we have found that about 98.5% of hereditary MTC are affected by a *RET* germline mutation but a few cases are still *RET* negative despite the screening covered all *RET* exons. To explain the hereditary nature of *RET* cases, the presence of a mutation in genes other than *RET* could be hypothesized or the presence of a *RET* intronic variant with the ability to modify *RET* expression levels could be considered [33].

In addition, and in keeping with other reports [18,34], we demonstrated the presence of a germline *RET* mutation in more than 6% of apparently sporadic MTC patients, thus confirming the important role of *RET* genetic screening for the identification of unsuspected MEN2 families [18]. Once an apparently sporadic MTC patient is reclassified as hereditary, *RET* genetic screening should be performed in all first-degree family members, allowing the identification of gene carriers who can thus benefit from appropriate screening, an early diagnosis and timely or prophylactic treatment measures.

In 1994 a study performed by the International *RET* Consortium (IRC) [35] analyzed 477 MEN 2 kindred and demonstrated that about 94% of cases presented with a *RET* germline mutation affecting one of the following codons: 609, 611, 618, 620, 634, 768, 804 and 918. In the present series, the percentage of *RET* positive families (193/195, 98.9%) was higher than that reported in the IRC study. This finding could be explained by the inclusion of a wider portion of the *RET* gene in the screen (to include additional variants identified since the publication of the IRC study), and perhaps to improvements in sequencing technologies. In fact, over the past 25 years, 197 different *RET* variants, (some pathogenic and others VUS), have been associated with MEN2 [16] and most of the cases that were originally considered negative for RET germline variants have been reanalyzed and reclassified as positive. Our wide *RET* screening approach identified 42 different point mutations. In our series, the most frequent *RET* mutation was the V804M in exon 14. Our findings support previously reported data [28,36] indicating that V804M is the most frequent *RET* variant associated with hereditary MTC in Italy, while mutations at codon 634 have been found to be the most frequent in Germany [37] and in other European and non-European countries [24,25]. The high prevalence of the V804M mutation in Italy is likely the result of a “founder” effect that amplified the prevalence of this specific, relatively uncommon mutation in the population.

The present study describes a different profile of *RET* germline mutations between the group of the clinically hereditary cases and the cohort of apparently sporadic cases. Although the V804M mutation is the most frequent single germline mutation observed in the two groups, mutations involving the cysteine at codon 634, and overall, mutations involving the cysteine residues in exons 10 and 11, are significantly more frequent in the group of clinically hereditary cases than in apparently sporadic cases. Conversely, mutations affecting non-cysteine residues are more frequent in apparently sporadic cases than in the clinically familial cases. This difference can be explained by the higher oncogenic potential and penetrance of the cysteine mutations, with particular regard to C634R, when compared to non-cysteine *RET* variants [15]. The different biological behavior of the mutations is responsible for a difference in the latency period and aggressiveness of the MTC and in the prevalence of the associated endocrine diseases (PHEO and hyperPTH) making the syndromes determined by cysteine mutations easier to be clinically identified [7,38].

Among all mutations, we found some rare variants whose biological significance is still unknown (VUS). The accurate characterization of the pathogenic role of these variants would be of great relevance for patients. The aggressiveness and the biological role of these new variants can be predicted by “in silico” on line tools [14,15] even if sometimes, the predicted results do not correlate with the in vitro assay. For this reason, clinicians should be cautious in trusting computational results to design appropriate treatment for cancer patients with these VUS. Among the 42 different *RET* germline mutations of our series, the Y791F mutation is still reported although recent studies indicate that this mutation does not increase the risk for MTC [39]. In agreement with these reported evidences, MTC was completely absent in one family with 3 positive members while in the other two only the index cases showed the MTC. Our interpretation is that in these latter two families, the index case had the MTC for other genetic reasons, likely for sporadic alterations, and the identification of the germline mutation was a “non-target” effect of the *RET* screening.

In our series the MEN2B patients were all affected by the M918T mutations in exon 16 of the *RET* gene that it is known to account for 95% of MEN2B cases [8,40]. As reported in other series [41], also in our cases the *RET* mutations in MEN2B were all de novo. In addition to the MEN2B cases, seven additional *RET* positive patients have been documented to be de novo and in these cases *RET* mutations were discovered in exon 10 or 11. The finding of de novo mutations in MEN2A is of interest and scarcely reported and discussed. Proving the de novo origin of these mutations would require the analysis of both parents and this is not always possible, especially in adult index cases whose parents may have already died. Moreover, since is not ethically acceptable to test for paternity, doubts about the progenitorship cannot be ruled out. However, it is conceivable that, as it happens for MEN2B [42], also in a subgroup of MEN2A and likely also of FMTC, the *RET* mutation happened as “somatic” event in one of the gametes or in the early phases of embryonic development.

In agreement with previous series [20,43], a strong genotype–phenotype correlation has been confirmed in our Italian cohort. In particular, the observation that MTC is strongly associated to *RET* mutations strengthens the idea that alterations in the *RET* gene are enough to induce tumoral transformation as indeed demonstrated by in vitro studies [44]. PHEO and/or hyperPTH are mainly associated with cysteine mutations, in particular at Cys634, and are very rare in families with non-cysteine *RET* mutations. In our series only three families with a non-cysteine *RET* mutation had members with PHEO or hyperPTH and were all harboring a V804M mutation. This evidence is relevant for clinicians because the follow up of patients with non-cysteine *RET* mutations could be simplified by eliminating the annual evaluations of adrenal gland and parathyroid function which are still recommended in the clinical practice guidelines. Of course, it remains mandatory in cases with cysteine mutations, particularly when a C634R is present.

Hirschsprung’s disease is also associated with *RET* mutations but, in contrast to those associated with MTC, they are inactivating mutations [45,46]. In some cases, mutations in *RET* C620, C618, C611 and C609, act as “Janus” mutations [47]: that are able to induce both Hirschsprung’s disease and MTC in the same patient or in the same family. These two diseases are present also in some members of three families of our series harbouring typical (cysteine-associated) *RET* “Janus” mutations. However, we have identified other two families with non-cysteine *RET* mutations, (K821E and Y791F) with one single member with Hirschsprung’s disease and, so far, no one with MTC. It is interesting that in both cases the mutations are in the intracellular domain of the receptor, in particular in exon 13 and 14, that are commonly screened for MTC but never demonstrated to be Janus mutation. Only the long-term follow up of these young patients and their *RET* positive relatives will clarify if they will ever develop MTC.

## 5. Conclusions

In this study, that to our knowledge is the largest series of screened MTC at a single center, we showed that: (a) *RET* genetic screening should be performed in all MTC cases independently from their clinical presentation; (b) V804M is confirmed to be the most frequent *RET* mutation in the Italian population; (c) cysteine mutations are more frequent in cases with a clinically manifested hereditary form; (d) the non-cysteine mutations are very rarely associated with PHEO and hyperPTH suggesting that the follow up of these patients could be simplified; (e) de novo mutations are frequent in MEN2B syndrome but they can be found also in both MEN2A and FMTC; (f) germline *RET* VUS may be found with genetic screening but their pathogenic role should be defined before testing relatives of the index case.

## Figures and Tables

**Table 1:** Variant of unknown significance: Prevalence in our series and in silico prediction of their transforming ability.

*RET* Mutation	Families	PATIENTS (n.)					In Silico Prediction					
		Studied	*RET* Positive	*RET* Negative	Affected	Gene Carriers	SIFT	POLYPHEN	CADD	REVEL	META LR	MUTATION ASSESSOR
**R215L ^a^**	1	1	1	0	1	0	tolerated	likely benign	likely benign	benign	tolerated	0.medium
**K666Q**	1	1	1	0	1	0	deleterious	possibly damaging	likely benign	Likely disease causing	damaging	low
**K821E**	1	3	3	0	1	2	deleterious	probably damaging	likely benign	Likely disease causing	damaging	low
**V871I**	1	4	3	1	1	2	deleterious	probably damaging	likely benign	Likely disease causing	damaging	neutral
**T338I**	1	3	2	1	1	1	tolerated	benign	likely benign	likely benign	tolerated	medium
**E632K**	1	1	1	0	1	0	tolerated	possibly damaging	likely benign	Likely disease causing	damaging	medium
**V648I**	2	6	2	4	1	1	tolerated	benign	likely benign	Likely disease causing	damaging	neutral
**R833C**	1	2	2	0	1	1	deleterious	probably damaging	likely benign	Likely disease causing	damaging	medium
**M848T**	1	2	2	0	1	1	tolerated	probably damaging	likely benign	Likely disease causing	damaging	neutral
**A883T**	2	14	9	5	3	7	deleterious	probably damaging	likely deleterious	Likely disease causing	tolerated	neutral

**^a^** this patient had a simultaneous C634Y somatic mutation.

**Table 2:** Prevalence of endocrine and non endocrine clinical phenotypes according to the type of mutation: Only mutations associated with multiple diseases are reported.

RET Mutation	Number of Families	Number of Families with PHEO/Number of Total Families	Number of Families with hyperPTH/Number of Total Families	Number of Families with Other Diseases/NUMBER of Total Families
V648I	3	0/3	0/3	1/3 ^a^
E768D	7	0/7	0/7	1/7 ^b^
V804M	50	2/50	1/50	12/50 ^a^
S891A	12	0/12	0/12	2/12 ^b^,1/12 ^d^
C609R	1	0/1	0/1	1/1 ^b^
C6111F	1	0/1	1/1	0/1
C618R	5	2/5	0/5	1/5 ^b^
C618S	5	1/5	0/5	0/5
C620R	4	1/4	0/4	1/4 ^a^
C620S	4	0/4	0/4	1/4 ^a^
C620Y	2	0/2	0/2	1/2 ^b^, 1/2 ^a^
C630Y	3	0/3	1/3	1/3 ^c, a^
C634F	3	3/3	0/3	1/3 ^c^
C634G	4	2/4	0/4	1/4 ^c^
C634R	20	13/20	3/20	2/20 ^c^
C634S	1	1/1	0/1	0/1
C634Y	15	11/15	3/15	1/15 ^c^
C634W	2	2/2	0/2	0/2
M918T	15	4/15	0/15	15/15

PHEO: pheochromocytoma; hyperPTH: hyperparathyroidism; ^a^ PTC; ^b^ Hirschprung’s disease; ^c^ Lichen cutaneous amyloidosic; ^d^ paraganglioma.
